# Serial Cardiovascular Magnetic Resonance Evolution of Late-Onset Female Danon Disease Initially Diagnosed as Hypertrophic Cardiomyopathy: A Case Report

**DOI:** 10.3390/jcm15156085

**Published:** 2026-08-05

**Authors:** Xuhan Liu, Shichu Liang, Jing Chen, Yucheng Chen

**Affiliations:** 1 Department of Cardiology, West China Hospital, Sichuan University, No. 37 GuoXue Alley, Chengdu 610041, Chinachenjing0014@163.com (J.C.); 2 Cardiac Imaging and Targets Therapy Lab, West China Hospital, Sichuan University, Chengdu 610041, China; 3 Center of Rare Diseases, West China Hospital, Sichuan University, Chengdu 610041, China

**Keywords:** Danon disease, *LAMP2*, cardiovascular magnetic resonance, hypertrophic cardiomyopathy phenocopy, bradyarrhythmia, case report

## Abstract

**Background/Objectives**: Danon disease is a rare X-linked lysosomal disorder caused by pathogenic variants in *LAMP2*. In women, cardiac involvement may occur later in life and may resemble sarcomeric hypertrophic cardiomyopathy (HCM), particularly when extracardiac manifestations are absent or subtle. A 42-year-old woman presented with chest discomfort in 2017 and was initially diagnosed with hypertrophic cardiomyopathy (HCM). She underwent serial 3.0-T cardiovascular magnetic resonance (CMR) over an 8-year period. Initial CMR showed left-ventricular hypertrophy, preserved left-ventricular ejection fraction (65.0%), increased native T1 and T2 relaxation times, extracellular volume (ECV) of 24.9%, and patchy apical late gadolinium enhancement (LGE extent, 12.35%). Five years later, worsening dyspnea was accompanied by increased left-ventricular mass index, higher native T1 and ECV, greater LGE extent (15.18%), and slow atrial fibrillation with ventricular ectopy on Holter monitoring. Genetic testing identified a likely pathogenic *LAMP2* variant, c.928G>A (p.Val310Ile), supporting the diagnosis of Danon disease in the clinical context. During a subsequent readmission three years later with acute amaurosis and dyspnea, no definite neurologic cause was identified in the available record. Repeat CMR showed the highest recorded native T1 and ECV values and diffuse LGE with relatively less interventricular-septal involvement, particularly in the basal septum (LGE extent, 24.59%); repeat Holter monitoring showed frequent long R-R intervals and ventricular escape beats. Because of progressive imaging and electrical deterioration, implantable cardioverter-defibrillator therapy and heart-transplantation assessment were recommended, and the patient ultimately chose to proceed with pre-transplant assessment in December 2025. **Conclusions**: Female LAMP2-related Danon disease may initially resemble HCM, but differs from it with diffusely abnormal T1/ECV measurements. Follow up in our case revealed progressive storage cardiomyopathy with diffuse myocardial injury and clinically relevant bradyarrhythmia.

## 1. Introduction

Danon disease is an X-linked dominant lysosomal and autophagy-related disorder caused by deficiency of lysosome-associated membrane protein 2 (LAMP-2) [1,2]. The classical syndrome includes cardiomyopathy, skeletal myopathy, cognitive impairment, and retinal disease, but the clinical expression is heterogeneous. In many patients, particularly women, cardiac involvement is the major determinant of prognosis and may be the first or only feature recognized in routine cardiology practice [3,4,5].

Female Danon disease is diagnostically challenging because the presentation is often delayed, extracardiac features may be subtle, and the initial phenotype may resemble HCM. Variable X-chromosome inactivation (lyonization) is considered one biological explanation for the broader and often later phenotype observed in heterozygous women, because the proportion of cardiomyocytes expressing the mutant allele may differ between individuals and tissues [3,4,6]. This overlap is clinically important because HCM remains a phenotypic diagnosis until relevant phenocopies have been excluded. A diagnosis of sarcomeric HCM may lead to conservative follow-up; whereas, Danon disease requires genetic counseling, systematic rhythm surveillance, family-oriented assessment, and timely discussion of device therapy and advanced heart-failure options [3,7].

CMR is useful when left-ventricular hypertrophy cannot be fully explained by routine clinical assessment. In addition to wall thickness and ventricular function, CMR provides information on LGE and quantitative tissue characteristics. Previous studies of Danon disease have described extensive LGE, frequent apical and free-wall involvement, relatively less interventricular-septal involvement, and abnormal T1 mapping [8,9,10,11,12]. The present case adds a longitudinal perspective. In a woman initially diagnosed as having HCM, serial 3.0-T CMR documented progression from apical-predominant injury to diffuse myocardial involvement with relatively less interventricular-septal involvement, followed by identification of a likely pathogenic *LAMP2* variant, worsening bradyarrhythmia, and eventual acceptance of pre-transplant assessment. The report was prepared according to the CARE guidelines; the completed CARE checklist is provided in the Supplementary Materials.

## 2. Case Presentation

### 2.1. Patient Information and Initial Assessment

A 42-year-old woman presented with chest discomfort in 2017 and was initially diagnosed with hypertrophic cardiomyopathy. The archived baseline record did not preserve a complete physical-examination note or detailed echocardiographic measurements from the time of the original diagnosis. The available baseline ECG report described no obvious abnormality and did not report pre-excitation; however, the original tracing and interval-level measurements were not preserved, so PR interval, voltage criteria for LVH, and subtle pre-excitation could not be independently re-evaluated. Baseline records did not document systematic screening for skeletal myopathy, cognitive impairment, or retinal symptoms. This incomplete phenotyping contributed to an HCM-oriented interpretation without specific evaluation for Danon disease at the initial presentation. No family history of Danon disease was documented, and a formal three-generation pedigree was not available in the retrospective record.

The original 2017 CMR report described left-ventricular enlargement, mild left-ventricular and interventricular-septal hypertrophy, a basal end-diastolic septal thickness of approximately 1.7 cm, preserved biventricular systolic function (left-ventricular ejection fraction, 65.0%; right-ventricular ejection fraction, 61.7%), reduced diastolic function, and patchy delayed enhancement. These findings were interpreted at that time as possible hypertrophic cardiomyopathy, pending correlation with other clinical tests. Subsequent review of the serial 3.0-T CMR dataset showed left-ventricular hypertrophy, increased native T1 (1344.5 ms) and T2 (48.9 ms) relaxation times, ECV of 24.9%, and patchy non-ischemic LGE mainly involving the left-ventricular apex (LGE extent, 12.35%). Mapping values were derived from global left-ventricular myocardial measurements on short-axis maps with blood pool and obvious artifacts excluded; the T1/ECV abnormality appeared more diffuse than the initially focal apical LGE. Left-ventricular mass index was 78.99 g/m^2^. The patient received beta-blocker and diuretic therapy and was followed clinically.

### 2.2. Follow-Up Evaluation and Genetic Diagnosis

Five years later, the patient developed worsening dyspnea. A 12-lead ECG in 2022 documented atrial fibrillation, ventricular premature beats, intraventricular conduction delay with QRS duration of 120 ms, ST-T changes, and a ventricular rate of 83 beats/min; no pre-excitation pattern was reported. The 2022 clinical CMR report described cardiomegaly dominated by biatrial enlargement, left-ventricular asymmetric hypertrophy with interventricular-septal predominance, maximal end-diastolic wall thickness of approximately 1.9 cm, relatively reduced myocardial motion, homogeneous first-pass myocardial perfusion, patchy delayed enhancement in the interventricular septum, mild mitral regurgitation, no pericardial effusion, and irregular cardiac rhythm that precluded reliable functional quantification, including LVEF. The original impression again considered hypertrophic cardiomyopathy possible and recommended correlation with clinical and other examinations. Subsequent serial CMR review demonstrated further myocardial thickening, increased left-ventricular mass index (82.88 g/m^2^), native T1 of 1358.4 ms, T2 of 46.5 ms, ECV of 29.5%, and more extensive subendocardial-to-mid-wall LGE involving the apical free wall (LGE extent, 15.18%). Mapping abnormalities remained diffuse relative to the LGE-positive regions. Holter monitoring showed atrial fibrillation with a slow ventricular response (average ventricular rate, 58 beats/min; fastest, 143 beats/min; slowest, 37 beats/min), a longest R-R interval of 2.05 s, three R-R intervals > 2.0 s per 24 h, multifocal ventricular premature beats (1448 per 24 h), paired ventricular premature beats (54 per 24 h), ventricular bigeminy, and three short non-sustained ventricular runs.

The combination of progressive hypertrophy, apical-predominant LGE, quantitative tissue abnormalities, and bradyarrhythmia was considered atypical for a stable sarcomeric HCM phenotype. Genetic testing was therefore performed and identified a likely pathogenic *LAMP2* variant, c.928G>A (p.Val310Ile), supporting the clinical diagnosis of Danon disease. This result reframed the clinical diagnosis from unexplained hypertrophy to an inherited lysosomal cardiomyopathy with implications for rhythm risk, family counseling, and advanced heart-failure planning. Genetic counseling and family assessment were advised. Heart-transplantation assessment was recommended, but the patient declined at that time and continued medical therapy.

### 2.3. Recent Deterioration

After three more years, during a subsequent readmission in 2025, the patient presented with acute amaurosis and dyspnea. The available neurologic evaluation did not identify a definite cause of the visual symptom. The medication list at the most recent evaluation included rivaroxaban, but the retrospective record did not allow confirmation of uninterrupted anticoagulation from 2022 to 2025. A 2025 ECG again showed atrial fibrillation and intraventricular conduction delay, with QRS duration prolonged to 140 ms, a ventricular rate of 75 beats/min, left-axis deviation (QRS axis, −92°), and ST-T changes. The 2025 clinical CMR report described cardiomegaly dominated by biatrial enlargement, mild asymmetric left-ventricular wall thickening with interventricular-septal predominance, maximal end-diastolic wall thickness of approximately 1.2 cm on the clinical report, relatively reduced myocardial motion, homogeneous first-pass myocardial perfusion, patchy delayed enhancement in the interventricular septum, myocardial mid-layer predominance, mild mitral regurgitation, no evident tricuspid regurgitation, no pericardial effusion, and irregular rhythm that again precluded reliable functional quantification, including LVEF. The original impression considered non-ischemic cardiomyopathy possible and recommended clinical correlation. Subsequent serial CMR review showed the highest left-ventricular mass index (92.48 g/m^2^), native T1 (1370.0 ms), and ECV (31.6%) values recorded during follow-up, and diffuse subendocardial-to-mid-wall LGE with relatively less interventricular-septal involvement, particularly in the basal septum (LGE extent, 24.59%). This pattern was consistent with previous CMR descriptions of Danon disease showing extensive enhancement with free-wall predominance and relatively less interventricular-septal involvement [8,10]. T2 relaxation time decreased to 43.2 ms, in contrast to the progressive T1, ECV, and LGE abnormalities, suggesting that chronic myocardial injury and replacement fibrosis, rather than acute edema, predominated in the later phase. A 2025 Holter recording showed atrial fibrillation with a slow ventricular response (average ventricular rate, 55 beats/min; fastest, 92 beats/min; slowest, 33 beats/min), 659 R-R intervals ≥ 2.0 s, a longest R-R interval of 2.990 s, and occasional ventricular escape beats. Implantable cardioverter-defibrillator therapy and renewed heart-transplantation evaluation were recommended. The patient ultimately chose to proceed with heart-transplantation assessment in December 2025.

### 2.4. Timeline

The major clinical, rhythm, cardiovascular magnetic resonance, and management milestones across the 8-year follow-up are summarized in Table 1.

## 3. Diagnostic Assessment

The diagnosis was revised only after the longitudinal findings were reviewed. At the first presentation, the patient was managed as having HCM because left-ventricular hypertrophy was present and systolic function was preserved, but in retrospect she had not undergone systematic evaluation for Danon disease-related musculoskeletal manifestations, cognitive features, retinal disease, or a formal family pedigree. During follow-up, several findings became difficult to reconcile with uncomplicated sarcomeric HCM: progressive myocardial thickening, LGE that was initially apical-predominant and later became diffuse with relatively less interventricular-septal involvement, worsening native T1 and ECV abnormalities, and clinically relevant bradyarrhythmia and conduction disturbance.

Genetic testing identified the *LAMP2* c.928G>A (p.Val310Ile) variant. The clinical genetic report classified the variant as likely pathogenic using an ACMG/AMP-based laboratory interpretation. Because the detailed criterion-level evidence table, family segregation analysis, and functional validation were not available in the retrospective record, the variant was interpreted together with the patient’s phenotype rather than as an isolated sequencing finding [6]. The cited Medicina report described a splice-affecting *LAMP2* variant, c.928+3A>G, in a Danon disease family and showed exon 7 skipping, frameshift, premature termination, and predicted loss of functional LAMP-2 protein [13]. Although that variant is not identical to the c.928G>A (p.Val310Ile) variant identified in the present patient, it supports the pathogenic relevance of disruption at this exon 7 splice region. Functional validation and family segregation analysis were not performed in the present patient. The serial CMR pattern, the development of rhythm disturbance, and the known relationship between *LAMP2* variants and Danon disease supported a clinical diagnosis of *LAMP2*-related cardiomyopathy.

The principal differential diagnoses included sarcomeric HCM, Fabry disease, mitochondrial cardiomyopathy, amyloidosis, myocarditis-related hypertrophic remodeling, Pompe disease and other glycogen-storage disorders, and additional storage or infiltrative cardiomyopathies. Several features argued against a simple sarcomeric HCM explanation: LGE progression from apical-predominant involvement toward a diffuse pattern with relatively less interventricular-septal involvement, the combination of high native T1 and ECV values, the free-wall emphasis, and the emergence of bradyarrhythmia. Fabry disease was less favored because the imaging phenotype did not show the typical low native T1 pattern expected in many untreated Fabry patients, and the genetic result identified *LAMP2* rather than *GLA.* Amyloidosis was not the leading diagnosis because the clinical course, age, hypertrophic pattern, and *LAMP2* result provided a more coherent explanation. Mitochondrial and other glycogen-storage cardiomyopathies can also cause hypertrophy and conduction abnormalities, but the serial CMR pattern together with a likely pathogenic *LAMP2* variant made Danon disease the most consistent final diagnosis. The diagnostic endpoint was therefore reached by integrating phenotype, serial imaging behavior, rhythm findings, and molecular testing.

No single CMR parameter was considered diagnostic by itself. The diagnosis depended on the combined pattern of wall thickness, LGE distribution, tissue-mapping abnormalities, rhythm findings, and genetic testing. This approach is important because mapping values are protocol-dependent; whereas, diffuse or expanding non-ischemic LGE with relatively less interventricular-septal involvement, worsening tissue characterization, and bradyarrhythmia provided a stronger signal of a phenocopy requiring molecular evaluation.

## 4. Therapeutic Intervention, Follow-Up, and Outcomes

Initial treatment consisted of beta-blocker and diuretic therapy. After the *LAMP2*-related diagnosis was made and disease progression became evident, the clinical team recommended heart-transplantation assessment. The patient declined transplantation evaluation at that stage and continued medical therapy.

At the most recent evaluation, symptoms, rhythm status, and CMR findings had all deteriorated. Long-term medical therapy included metoprolol succinate 23.75 mg once daily, rivaroxaban 10 mg once daily, spironolactone 10 mg once daily, furosemide 10 mg once daily, and potassium chloride sustained-release tablets 1000 mg three times daily, with alprazolam as needed and quetiapine at night. The available medication record confirmed rivaroxaban at the most recent evaluation but did not establish whether anticoagulation had been uninterrupted since the onset of atrial fibrillation in 2022. The marked increase in long R-R intervals, ventricular escape beats, and widening QRS duration strengthened the concern that electrical disease was progressing in parallel with myocardial injury. The team recommended moving beyond symptomatic medical therapy to implantable cardioverter-defibrillator therapy and advanced heart-failure evaluation. The patient ultimately chose to proceed with heart-transplantation assessment in December 2025.

## 5. Discussion

The diagnostic difficulty in this patient was the initially isolated hypertrophic phenotype and the incomplete early evaluation for Danon disease. The patient was initially regarded as having HCM, but the retrospective baseline record did not show that musculoskeletal manifestations, cognitive features, retinal disease, or a formal family pedigree had been systematically assessed. The available baseline ECG report did not report pre-excitation or another overt abnormality, but the original tracing and interval-level measurements were not available for independent review. The diagnosis was questioned only after follow-up CMR demonstrated progressive myocardial injury and rhythm monitoring showed atrial fibrillation with a slow ventricular response, followed by frequent long R-R intervals and ventricular escape beats. The *LAMP2* result then provided a plausible unifying explanation for the imaging and rhythm findings.

The case also shows why the absence of a classic multisystem presentation should not exclude Danon disease. Female patients may present later than male patients and may have less obvious extracardiac involvement at the time of cardiac evaluation. Male patients more often show a hypertrophic phenotype; whereas, heterozygous female patients may show either hypertrophic or dilated cardiomyopathy phenotypes [3,4,14]. Variable X-chromosome inactivation provides a plausible biological explanation for this heterogeneity and may contribute to delayed or incomplete presentation in heterozygous women [3,4,14]. However, later onset does not imply a benign cardiac course. In the JAMA *LAMP2* cardiomyopathy cohort, affected patients experienced progressive heart failure, sudden death, aborted cardiac arrest, and transplantation [7]. Registry and natural-history studies have similarly shown that women remain at risk for major cardiac events [4,5,14].

Longitudinal observation has also been useful in other inherited metabolic cardiomyopathies. Gragnaniello and colleagues described a patient with Pompe disease in whom a hypertrophic phenotype evolved toward non-compaction during follow-up [15]. Although Pompe disease and Danon disease are distinct entities, both examples support the same clinical point: in storage cardiomyopathies, a changing myocardial phenotype may be more informative than a single baseline study.

**Figure 1:**
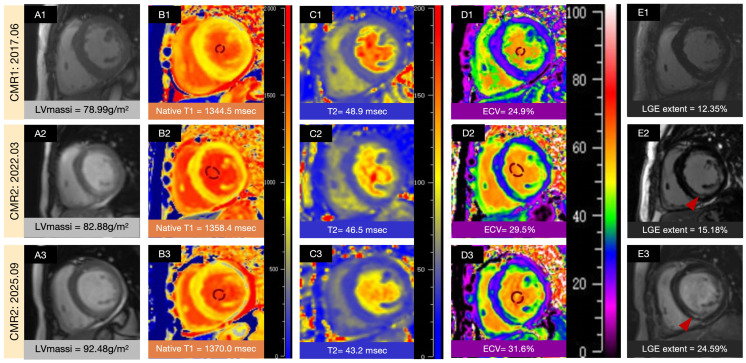
Serial cardiovascular magnetic resonance findings over 8 years in female Danon disease. (**A1**–**A3**), cine images showing progressive left-ventricular hypertrophy with increasing left-ventricular mass index. (**B1**–**B3**), native T1 maps showing progressive global T1 abnormality. (**C1**–**C3**), T2 maps showing decreasing T2 relaxation times during follow-up. (**D1**–**D3**), extracellular volume maps showing progressive expansion of abnormal extracellular volume. (**E1**–**E3**), late gadolinium enhancement images showing progression from patchy apical enhancement to diffuse subendocardial-to-mid-wall enhancement with relatively less interventricular-septal involvement, particularly in the basal septum, and increasing measured LGE extent. Red arrowheads highlight representative areas of LGE. CMR, cardiovascular magnetic resonance; ECV, extracellular volume; LGE, late gadolinium enhancement.

CMR was central to the diagnostic reassessment. Earlier multicenter CMR work described frequent apical and free-wall involvement, extensive subendocardial enhancement, and relative mid-basal septal sparing in Danon disease [8]. In a Chinese family report, the most common LGE location was the LV free wall; whereas, interventricular-septal involvement was less frequent, and native T1 was reported to be elevated even in regions without LGE [10]. Family-based CMR reports, T1-mapping studies, and reports of atypical LGE patterns have also supported the role of tissue characterization [10,12,16,17]. In the present patient, LGE extent increased from 12.35% to 24.59%, native T1 from 1344.5 ms to 1370.0 ms, ECV from 24.9% to 31.6%, and left-ventricular mass index from 78.99 to 92.48 g/m^2^ (Figure 1). The tissue-mapping abnormalities were global myocardial measurements and were not limited to LGE-positive regions. This serial pattern was more consistent with progressive storage-related myocardial injury than with stable sarcomeric HCM, particularly because the later study showed diffuse subendocardial-to-mid-wall LGE with relatively less interventricular-septal involvement.

The mapping results should be interpreted cautiously. All CMR examinations were performed at 3.0 T, but native T1, T2, and ECV values remain dependent on local acquisition and post-processing methods. For this reason, the absolute values were not treated as universal diagnostic thresholds. The more relevant finding was the within-patient trend: increasing left-ventricular mass index, expanding LGE, and worsening global T1/ECV abnormalities. A diffusely elevated native T1 and ECV at the first CMR were already atypical for uncomplicated HCM and, in retrospect, represented an early clue to an HCM phenocopy. The later decrease in T2 suggested that chronic myocardial injury and fibrosis, rather than persistent edema, dominated the later phase.

The rhythm findings added clinical urgency. Danon disease has been associated with atrial arrhythmias, ventricular arrhythmias, and conduction disease [3,4,5,7]. Previous work reported a relationship between fragmented QRS complexes, left-ventricular fibrosis, and function in Danon disease [18]. The available baseline ECG report described no obvious abnormality and did not report pre-excitation, but the original tracing and interval-level measurements were not preserved. The 2022 ECG documented atrial fibrillation, ventricular premature beats, intraventricular conduction delay with QRS duration of 120 ms, and ST-T changes, without reported pre-excitation. The 2022 Holter recording showed slow atrial fibrillation with three R-R intervals > 2.0 s per 24 h and multifocal ventricular ectopy. By 2025, the ECG again showed atrial fibrillation and intraventricular conduction delay, with QRS duration prolonged to 140 ms, left-axis deviation, and ST-T changes; Holter monitoring showed 659 R-R intervals ≥ 2.0 s, a longest R-R interval of 2.990 s, and occasional ventricular escape beats. This single case cannot establish a causal relationship between myocardial fibrosis and the slow ventricular response or ventricular escape rhythm. Nevertheless, the temporal association between increasing LGE burden and worsening rhythm abnormalities supported closer rhythm surveillance. The episode of acute amaurosis was also interpreted cautiously. No definite neurologic cause was identified in the available record, and the mechanism remains uncertain. Although rivaroxaban was listed at the most recent evaluation, uninterrupted anticoagulation from 2022 to 2025 could not be confirmed retrospectively; therefore, the event should not be overinterpreted mechanistically.

The diagnosis also changed management priorities. No established therapy reliably reverses Danon cardiomyopathy in routine clinical practice. After the *LAMP2*-related diagnosis was made, follow-up needed to include genetic counseling, family assessment when appropriate, repeated rhythm monitoring, reassessment of scar burden, and early referral to inherited cardiomyopathy and advanced heart-failure teams [3,11,19]. In this patient, transplantation assessment and implantable cardioverter-defibrillator therapy were recommended because symptoms, imaging findings, atrial fibrillation with slow ventricular response, frequent long R-R intervals, ventricular escape beats, and QRS widening all suggested disease progression.

Serial imaging may also help patient communication. For a patient previously followed as having HCM, recommendations for device therapy or transplantation assessment may appear abrupt. Showing the progression of myocardial injury on CMR can help explain why the diagnosis and management plan have changed.

The main limitation is the single-patient design, which precludes conclusions about prognosis or treatment efficacy. Baseline physical-examination findings, detailed baseline echocardiographic measurements, the original baseline ECG tracing with interval-level measurements, and a formal three-generation pedigree were not available in the retrospective record. Although the serial CMR examinations were performed at 3.0 T on Siemens scanners with a consistent protocol and Medis post-processing, mapping values remain dependent on scanner platform, acquisition sequence, and post-processing methods. Reliable LVEF measurements were available from the 2017 CMR examination, but irregular rhythm precluded reliable functional quantification in the 2022 and 2025 clinical CMR reports. The detailed ACMG criterion-level evidence table, family segregation analysis, myocardial histology, and functional validation of the *LAMP2* variant were not available. Although the patient underwent pre-transplant assessment in December 2025, subsequent transplant-listing status and longer-term outcome were unavailable when the manuscript was prepared. These limitations should be considered when interpreting the case, but they do not change the main diagnostic message: diffuse or expanding non-ischemic LGE with relatively less interventricular-septal involvement, worsening tissue characterization, and rhythm deterioration in a woman with an HCM phenotype should raise consideration of LAMP2-related Danon disease.

## 6. Patient Perspective

The patient was informed that the imaging, rhythm findings, and genetic results supported Danon cardiomyopathy rather than sarcomeric hypertrophic cardiomyopathy. According to the clinical record, she was concerned about the seriousness of the revised diagnosis and initially preferred continued medical treatment rather than transplantation assessment. After the subsequent deterioration, the clinical team again discussed device therapy and advanced heart-failure evaluation, emphasizing that these recommendations reflected progressive myocardial disease rather than a single isolated test result. The patient subsequently chose to proceed with pre-transplant assessment in December 2025.

## 7. Conclusions

Female LAMP2-related Danon disease may initially resemble HCM, but differs from it with diffusely abnormal T1/ECV measurements. Follow up in our case revealed progressive storage cardiomyopathy with diffuse myocardial injury and clinically relevant bradyarrhythmia.

## Figures and Tables

**Table 1:** Clinical, rhythm, cardiovascular magnetic resonance, and management timeline.

Time Point	Clinical Event	Rhythm Findings	CMR Findings	Management
2017 baseline	Chest discomfort; initial diagnosis of hypertrophic cardiomyopathy	Available ECG report: no obvious abnormality or reported pre-excitation; tracing/interval measurements unavailable	3.0-T CMR: LVEF 65.0%; LVMi 78.99 g/m^2^; T1 1344.5 ms; T2 48.9 ms; ECV 24.9%; apical LGE 12.35%	Beta-blocker and diuretic therapy
5-year follow-up	Worsening dyspnea	ECG/Holter: AF, QRS 120 ms; mean 58 bpm; R-R > 2.0 s x3; longest 2.05 s; multifocal PVCs; short ventricular runs	3.0-T CMR: LVEF not reliably quantified because of irregular rhythm; LVMi 82.88 g/m^2^; T1 1358.4 ms; T2 46.5 ms; ECV 29.5%; apical/free-wall LGE 15.18%	*LAMP2* testing; genetic counseling/family assessment advised; transplantation assessment declined
2025, three years later	Acute amaurosis and dyspnea	ECG/Holter: AF, QRS 140 ms; mean 55 bpm; R-R >= 2.0 s x659; longest 2.990 s; escape beats	3.0-T CMR: LVEF not reliably quantified because of irregular rhythm; LVMi 92.48 g/m^2^; T1 1370.0 ms; T2 43.2 ms; ECV 31.6%; diffuse LGE 24.59% with relatively less IVS involvement	Rivaroxaban listed at most recent evaluation; uninterrupted anticoagulation since 2022 not confirmed; ICD therapy recommended; pre-transplant assessment chosen in Dec 2025

Note: CMR values are reported from serial 3.0-T Siemens examinations analyzed with Medis software. Local 3.0-T reference ranges from recent institutional CMR reference data were native T1, 1112–1292 ms, and T2, 30–41 ms; ECV and LGE extent are reported as measured values rather than universal diagnostic thresholds. Mapping values represent global left-ventricular myocardial measurements rather than measurements restricted to LGE-positive segments. AF, atrial fibrillation; CMR, cardiovascular magnetic resonance; ECV, extracellular volume; ICD, implantable cardioverter-defibrillator; LGE, late gadolinium enhancement; LVEF, left-ventricular ejection fraction; LVMi, left-ventricular mass index; PVCs, premature ventricular complexes.

## Data Availability

The data supporting this case report are available from the corresponding author on reasonable request, subject to patient privacy and institutional regulations.

## Supplementary Materials

The following supporting information can be downloaded at: https://www.mdpi.com/article/10.3390/jcm15156085/s1, Video S1: 2017cine_1.1. Video S2: 2022cine_1.1; Video S3: 2025cine_1.1; Table S1: CARE checklist.
