# Supplementary material for: Serial Cardiovascular Magnetic Resonance Evolution of Late-Onset Female Danon Disease Initially Diagnosed as Hypertrophic Cardiomyopathy: A Case Report
Source: J Clin Med. 2026 Aug 5;15(15):6085. doi: 10.3390/jcm15156085 (PMC13466649; doi:10.3390/jcm15156085)
Supplement: Supplementary file 1 [file jcm-15-06085-s001.zip › jcm-4396609-Table S1.pdf]

CARE Checklist for Case Report Submission

Manuscript title: Case Report: Eight-Year Cardiovascular Magnetic Resonance Evolution in Late-Onset Female Danon Disease With Bradyarrhythmia

This checklist was prepared according to the 2013 CARE case report reporting guideline.

Topic	Item	Checklist item	Reported in manuscript
Title	1	The words case report are included in the title, together with the area of focus.	Title page
Keywords	2	Two to five keywords identify the diagnosis or intervention, including case report.	Keywords
Abstract	3a	What is unique about the case and what it adds to the literature.	Abstract, Background
Abstract	3b	The patient's main concerns and important clinical findings.	Abstract, Case presentation
Abstract	3c	The primary diagnosis, interventions, and outcomes.	Abstract, Case presentation
Abstract	3d	The main take-away lesson of the case report.	Abstract, Conclusion
Introduction	4	Brief background and why the case is unique, with relevant literature.	Introduction
Patient Information	5a	De-identified patient-specific information.	Case Description
Patient Information	5b	Primary concerns and symptoms.	Case Description; Timeline
Patient Information	5c	Medical, family, and psychosocial history including relevant genetic information.	Case Description; Diagnostic Assessment
Patient Information	5d	Relevant past interventions and their outcomes.	Case Description; Therapeutic Intervention
Clinical Findings	6	Relevant physical examination and other clinical findings.	Case Description; Diagnostic Assessment
Timeline	7	Important dates and times summarized as a timeline.	Timeline table
Diagnostic Assessment	8a	Diagnostic methods, including imaging, Holter monitoring, and genetic testing.	Diagnostic Assessment
Diagnostic Assessment	8b	Diagnostic challenges and reasoning.	Diagnostic Assessment; Discussion
Diagnostic Assessment	8c	Diagnosis and differential diagnosis.	Diagnostic Assessment
Diagnostic Assessment	8d	Prognostic characteristics when applicable.	Discussion
Therapeutic Intervention	9a	Types of therapeutic intervention.	Therapeutic Intervention
Therapeutic Intervention	9b	Administration and changes in intervention.	Therapeutic Intervention
Follow-up and Outcomes	10a	Clinician-assessed and patient-assessed outcomes.	Follow-up and Outcomes; Patient Perspective
Follow-up and Outcomes	10b	Important follow-up diagnostic and intervention outcomes.	Follow-up and Outcomes
Follow-up and Outcomes	10c	Intervention adherence and tolerability.	Therapeutic Intervention; Follow-up and Outcomes
Follow-up and Outcomes	10d	Adverse and unanticipated events.	Case Description; Follow-up and Outcomes
Discussion	11a	Strengths and limitations of the approach to this case.	Discussion
Discussion	11b	Relevant medical literature.	Introduction; Discussion
Discussion	11c	Rationale for conclusions.	Discussion
Discussion	11d	Primary take-away lessons.	Discussion; Conclusion
Patient Perspective	12	Patient perspective or explanation if unavailable.	Patient Perspective
Informed Consent	13	Written informed consent was obtained for publication.	Ethics Statement
